# Preventive Effectiveness of Thoracic Side Airbags in Side-Impact Crashes Based on Korea In-Depth Accident Study (KIDAS) Database

**DOI:** 10.3390/ijerph192315757

**Published:** 2022-11-26

**Authors:** Joon Seok Kong, Kang Hyun Lee, Chan Young Kang, Dooruh Choi, Oh Hyun Kim

**Affiliations:** 1 Center for Automotive Medical Science Institute, Wonju College of Medicine, Yonsei University, Wonju 26426, Republic of Korea; 2 Department of Emergency Medicine, Wonju College of Medicine, Yonsei University, Wonju 26426, Republic of Korea

**Keywords:** thorax injury, thoracic side airbags, side-impact, rib fracture, motor vehicle crashes, trauma center, Korea In-Depth Accident Study (KIDAS)

## Abstract

Studies on the effectiveness of thoracic side airbags (tSABs) in preventing thoracic injuries is limited and conflicting. This retrospective observational study aims to evaluate the effectiveness of tSABs in side-impact crashes based on data for motor vehicle occupants (MVOs) who visited an emergency department in Korea. The data were obtained from the Korean In-Depth Accident Study (KIDAS) database for patients treated at Wonju Severance Christian Hospital between January 2011 and April 2020. Of the 3899 patients with road traffic injuries, data for 490 patients were used. The overall frequency of tSAB deployment in side-impact crashes was found to be 8.1%. In the multivariate analysis, elderly age, near-side impact, colliding with fixed objects, non-oblique force, and higher crush extent were found to be factors associated with higher thoracic injuries (Abbreviated Injury Scale ≥ 2). MVOs in crashes with tSAB deployment were at an increased risk of injury compared with MVOs in crashes with no deployment, but no statistical difference was observed [adjusted odds ratios (AORs): 1.65 (0.73–3.73)]. Further, the incidence of lung injury and rib fractures increased with tSAB activation (*p* < 0.05). These results demonstrate the limited capability of tSABs in preventing thoracic injuries in motor vehicle crashes.

## 1. Introduction

In motor vehicle crashes (MVCs), side impact poses a relatively higher risk of severe injuries and fatalities than frontal and rear impacts [1]. In 2018, side-impact occupants accounted for 23% of fatalities - currently the second most catastrophic type of MVC in the United States [2]. A previous study from Australia reported that side-impact crashes accounted for 25% of all injury crashes and 40% of serious injury crashes where an MVO was either hospitalized or killed [3]. The national datasets of UK (The British National Accident Database, STATS 19), France (Bulletin d’Analyse des Accidents Corporels de la Circulation, BAAC), and Sweden (Swedish Traffic Accident Data Acquisition, STRADA) contain a total of 411,311 crashes, with the side-impact crashes typically representing 33% of all fatalities [4]. Side-impact crashes are accounted as most common crashes is some countries, causing serious concerns. In China, more than 26% of road traffic crash deaths occurred in side-impact crashes (25%) followed by frontal crashes (26%). This was nearly twice the amount of higher occurrence compared to MVCs with a fixed object or pedestrian (17%) and rear-end crashes (14%) [5]. Side-impact crashes are the most common on Polish roads, accounting for at least twice the frontal and rear-end crashes [6]. Compared with front- and rear-end crashes, the impact in a side-impact crashes are transmitted relatively intensively to the occupant compartment and the capacity to absorb impact energy is limited [7]. Thus, in real-world side-impact crashes, motor vehicle occupants (MVOs) are at a higher risk of suffering from serious injuries [8].

Passive safety devices play a significant role in protecting MVOs from side impact [9,10,11]. Although modern seatbelt designs are the same (three-pointed across lap that should be fastened), airbag systems have been developed in various forms depending on the collision direction. Compared to frontal airbags, the side airbag (SAB) is a relatively new passive safety system initially introduced in certain models by Volvo in 1995. These airbags are fabric-based and actively operate in case of a crash by using an electronic control system that triggers inflating chemical agents. Airbags installed in doors or seats are designed to damp and redistribute forces exerted on the head, chest, and/or abdomen by the intruding side of a vehicle [12,13]. SABs are classified into different types based on the target body part owing to changes in geometry: roof rail-embedded (or inflatable curtain) head airbags (hSABs), door or seat-mounted thoracic side airbags (tSABs), and (the more recently developed) combination airbags deployed from the seatback (provides protection for the head as well as the torso) [14].

The chest and head are the most commonly affected regions in side-impact injuries. In particular, the chest is known to have the highest risk of serious injuries [8,15,16]. However, while data in the literature are enough to prove the effectiveness of SABs in preventing injuries in the real world, it is insufficient to draw statistically significant conclusions [13]. Moreover, conflicting results regarding the effectiveness of SABs have been reported depending on their type [17]. In a previous study, combination head and torso airbags were reported to significantly reduce the risk of death in MVOs by 45% and 37%, respectively [18,19]. McGwin et al. reported that vehicles equipped with hSAB had a 75% lower risk of head injury in near-side impacts [20]. In addition, a similar inflatable tubular hSAB has been reported to significantly reduce head injury [18].

In terms of thoracic injuries, side airbags (seat- and door-mounted, inflatable tubular, and curtains) that provide thorax protection have been reported to reduce the risk of injuries by 68% [20]. However, compared with the combination SAB, the torso-only SAB was found to be generally less effective in reducing driver mortality [18,19]. Meanwhile, Gaylor and Junge [9] used the German In-Depth Accident Study (GIDAS) database to argue that tSAB had no effect in reducing severity of chest injuries [14]. A single deployed tSABs showed no ability to prevent thoracic injury severity and rib fractures in MVOs [21,22]. However, only a few studies have analyzed the effects of tSABs by delving into the detailed diagnosis of clinical outcomes. In addition, previous studies have limitations such as the absence of a detailed diagnosis or the existence of undiagnosed fractures, because they focused on the Abbreviated Injury Scale (AIS) or their diagnosis was limited to X-ray scans.

Thus, the aim of this study was to evaluate the effectiveness of tSAB deployment for preventing thoracic injuries, along with injury severity and detailed diagnosis of injuries in side-impact crashes (including near- and far-side) using Emergency Medical Record (EMR) data from a regional trauma center in South Korea.

## 2. Methods

### 2.1. Data Source

This study included trauma patients registered in the Korea In-Depth Accident Study (KIDAS) database covering medical records at Wonju Severance Christian Hospital. The data collection in KIDAS is performed by medical staff for injury surveillance and documents crash-related information such as vehicles involved and road environments. We analyzed MVOs who visited a single regional trauma center between January 2011 and April 2021. The data refer to the crash report based on real-world investigations of aspects such as seatbelt usage, airbag deployment, seat position, impact directions, collision objects, and vehicular crash deformations at the time of crash. The variables in the database refer to an interdisciplinary depth of details to configure the presence of MVCs related to traumas in South Korea. This study was conducted following approval from the research ethics committee of the Wonju Severance Christian Hospital at Yonsei University (IRB Approval No.: CR317137).

### 2.2. Study Population

**Figure 1:**
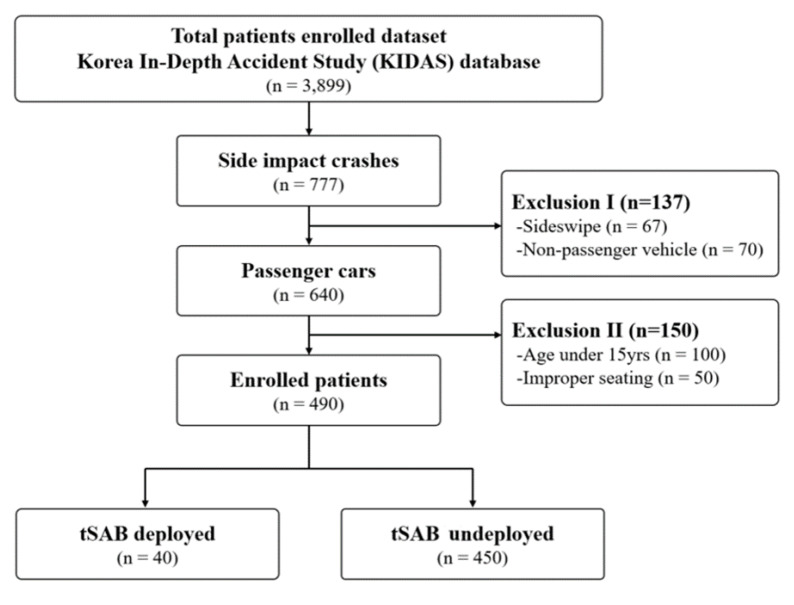
Flowchart of study participants. KIDAS; Korea In-Depth Accident Study, tSAB; thoracic side airbags.

This was a retrospective observational study wherein we selected 490 patients aged ≥ 15 years who were admitted to the emergency department (See Figure 1). We only considered occupants who were seated in the first and second rows of passenger cars (i.e., sedans, sports-utility vehicles, and vans). In terms of side impacts, multiple crashes and sideswipes were excluded from this study. Age criteria were divided into elderly (65 years old and above) and non-elderly groups.

### 2.3. Crash Characteristics 

**Figure 2:**
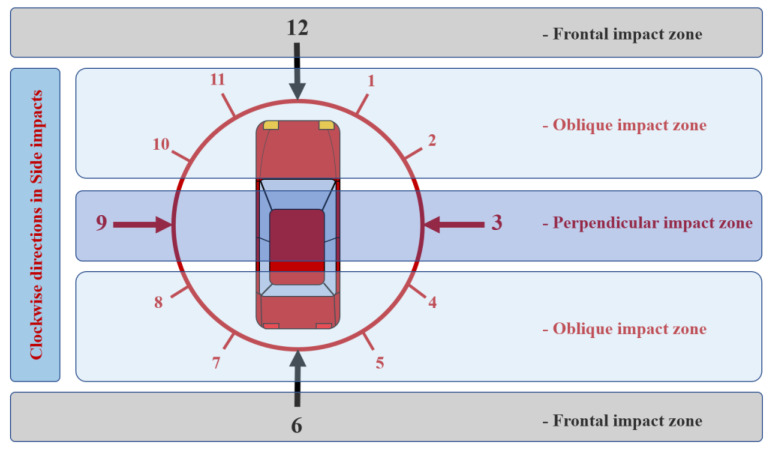
Clockwise directions in side impacts.

Side impact was selected for 1–5 and 7–11 clockwise directions using the principal direction of force (Figure 2). In addition, we redefined the force direction with the impact direction, which was classified into perpendicular (3 and 9 clockwise) and oblique impacts (1–2, 4–5, 7–8, and 10–11 clockwise). The impact direction was classified into near- and far-side impacts according to the seating position of the occupant and crash position. In this study, the effectiveness of tSABs in protecting against thoracic injuries were analyzed considering only seat-mounted side airbags. In the case of colliding partners, vehicles were described as movable objects, whereas guard rails, walls, trees, and poles were defined as fixed objects. Crash severity was determined using the Collision Deformation Classification (CDC) codes issued by the Society of Automotive Engineers (SAE) International [23]. We classified crush extent (CE) ≥ 2 as severe crashes with expected internal intrusions.

### 2.4. Binary Injury Severity Classification 

As mentioned previously, the injury severity of MVOs was determined using AIS [24], which is a medical index that is widely used as a grading scale according to the site of injury in traumatology. In this study, we evaluated the effectiveness of tSAB deployment using thoracic-AIS2+. Furthermore, the injury prevention capability of tSABs was investigated using detailed diagnostic details for thoracic injuries in five regions (diaphragm injury, lung contusion, hemothorax, pneumothorax, rib fracture, and sternum fracture).

### 2.5. Evaluation of Preventive Effectiveness 

In clinical and epidemiologic research, calculating adjusted odds ratios (AORs) with confidence interval (CI) for continuous covariates is used for logistic regression. The odds ratio (OR) can also be used to determine whether a particular exposure is a risk for a specific outcome and to compare the magnitude of various risk factors for those outcomes. The exponential function of the regression coefficient is the OR associated with a one-unit increase in the exposure. AORs measure the association between a cofounding variable and the outcomes and controls for that value. A Hosmer-Lemeshow goodness-of-fit test was performed to determine the fitness.

### 2.6. Statistical Analysis 

The study population was divided into two groups according to the tSAB deployments. Categorical variables were expressed as counts and proportions, and continuous variables were expressed as median and interquartile range (IQR). Differences between the two groups were compared using Pearson’s chi-square test and Student’s *t*-test. AORs with 95% CI of exposure variables using endpoints were calculated using multivariate logistic regression analysis with reference. The model was adjusted for age, seatbelt use, tSAB deployment, seat position, side impacts, collision partner, impact direction, and crush extent, which had statistical significance to thoracic-AIS2+ injuries. We considered the potential effectiveness of tSABs on both the overall side impact and individual conditions (near- and far-side impacts). The criterion for the *p*-value was defined as a two-sided significance level of 0.05. All statistical analyses were performed using SPSS Statistics for Windows, version 26.0 (IBM Corp., Armonk, NY, USA).

## 3. Results

This study analyzed the preventive effectiveness of thoracic injury risk associated with tSAB deployment in side-impact crashes. Table 1 shows the demographic characteristics of MVOs according to tSAB availability during a side impact. The overall frequency of SAB deployment in the MVOs was 8.1%. In this study, tSAB deployment was higher in belted occupants than in unbelted occupants MVOs (*p* = 0.006). The product manufacturing years of the vehicle used began in 2005, whereas most tSAB-deployed crashes were counted from 2010 or later (*p* < 0.001). The near-side impact had a higher tSAB deployment rate than the far-side impact (*p* = 0.007). However, tSAB deployment was not statistically affected by sex, age, height, weight, BMI, seat position, vehicle type, collide partner, PDOF, and crush extent. In addition, it is difficult to judge whether the deployment of tSAB affected the severity of thoracic injury.

A multivariate logistic regression was performed to analyze the risk factors of thoracic injury severity of MVOs associated with adjusted ORs for age group, seat belt usage, tSAB deployment, seating position, collide partner, and crush extent (See Table 2). Overall, elderly occupants showed higher injury risks than non-elderly occupants in side impacts (AOR: 2.96 [1.33–6.73]). Moreover, compared with far-side impacts, occupants in near-side impacts showed significantly higher risk of AIS2+ injuries (AOR: 4.30 [2.33–7.94]). In the case of collision partners, colliding with fixed objects was found to be more common than car-to-car crashes (AOR: 2.57 [1.26–5.26]). Furthermore, oblique impacts showed a lower risk of thoracic injuries compared with perpendicular impacts (AOR: 0.43 [0.25–0.76]). On the contrary, the risk of thoracic injury was mostly threatened by an increase in crush extent (AOR: 4.85 [2.69–8.74]). However, passive safety systems (including seatbelts and tSABs) showed no effectiveness in preventing thoracic injuries.

Furthermore, the study segregated the overall side impact into near-sided and far-sided impacts to analyze the thoracic injury mechanism. The risk of thoracic injury was highly significant in both near-side (AOR: 4.58 [2.27–9.21]) and far-side (AOR: 6.15 [1.87–20.25]) impacts - influenced by the increase in crush extent. Specifically, risk of thoracic injury was critical in the case of elderly MVOs (AOR: 3.41 [1.12–10.36]), collision with fixed objects (AOR: 2.95 [1.24–7.02]), and second-row MVOs (AOR: 4.24 [1.34–13.47]), respectively. However, injury risk reduced in oblique impacts compared with perpendicular ones (AOR: 0.42 [0.26–0.84]). Regardless of both near- and far-sided impacts, tSABs had no statistically significant effect on preventing thoracic injuries.

This study also examined the statistical significance of the detailed thoracic regions according to whether tSAB was deployed in side-impact crashes (Table 3). Consequently, it was found that the risk of injury increased during tSAB deployment in most regions (lungs, hemo- and pneumo-thorax, ribs, and sternum). In particular, compared with the undeployed tSAB case, occupants with deployed tSAB had increased risk of lung and rib fractures. The hemothorax, pneumothorax, and sternum fractures also had higher rates of incidence in case of tSAB activation; however, there was no statistical difference.

Subsequently, multiple logistic regression analysis was performed, in which the previously identified major regions of AIS2+ thoracic injuries were analyzed (See Table 4). In the case of lung contusions and lacerations, the higher risk of AIS2+ injury was affected by the increase in crush extent (AOR: 3.50 [1.56–7.85]). In contrast, the risk of rib fractures had an increasement in near-side impacts (AOR: 2.97 [1.51–5.85]) and CE2+ (AOR: 5.07 [2.50–10.28]), respectively. However, compared with perpendicular side impacts, the injury risk was reduced in oblique side impacts (AOR; 0.46 [0.25-0.86]). Nevertheless, the deployment of tSABs was not statistically effective in preventing both lung injury (AOR: 2.35 [0.92–6.03]) and rib fractures (AOR: 1.99 [0.88–4.67]).

## 4. Discussion

In this study, tSABs were found to be ineffective in preventing thoracic injuries in MVOs involved in with side-impact crashes. Many previous studies have also reported that the risk to the thoracic region was similar regardless of tSAB deployment [21,22,25,26]. Some researchers suggested that SABs do not contribute to injury prevention [16,17]. On the contrary, others provide a statistical difference in the deployment of different types of SABs that had preventive effects in side impacts [27]. There are still many controversies on the effectiveness of tSAB; however, our study showed no effect in reducing thoracic injuries. 

In this study, the factors affecting the presence of AIS2+ thoracic injury in a side-impact crashes were elderly age, near-side impact, collision with fixed objects, perpendicular impact, and CE2+. It was already known that the risk of AIS2+ thorax injury increased in the elderly involved in side-impact crashes. Numerous studies have reported that aging increases the injury severity in patients with MVCs. These studies support similar results in that elderly occupants sustained a higher incidence of thorax injury than non-elderly occupants [28]. Furthermore, Page et al. suggested that aging increases injury risks in all types of side impacts [17]. Our study showed some relative results regarding elderly MVOs shows higher risk of thoracic injuries in side impacts. 

Compared with far-side impacts, the presence of AIS2+ thoracic injuries was higher in near-side impacts. This is because the collision energy is directly applied to MVOs located adjacent to the crash direction. This is a similar result regarding the contribution of near-side impacts to serious injuries in many previous studies [29,30,31,32,33]. In particular, the probability of thorax injury in near-side impacts was significantly higher than that in far-side impacts [34,35,36]. 

In this study, colliding with fixed-material objects was suggested to be a major risk factor affecting the presence of thoracic injuries. In the case of car-to-car crashes, it is possible to reduce the risk of injury to passengers owing to crash compatibility through absorption of collision energy between two vehicles. However, in colliding with fixed objects, the impact of the collision force tends to be greater on MVOs. In particular, colliding with objects with a relatively narrow range of structural volume (such as a pole or a tree) may cause serious crash severity, as the impact force is centralized in the contact area. 

In this study, the risk of AIS2+ thoracic injury increased in perpendicular side impacts compared with that for oblique crashes. In previous studies related to impact direction, more than half of fatal crashes occurred during perpendicular side impacts [37,38,39,40]. A relatively wide range of collision deformations on the vehicular body may force larger collision energy on MVOs. Thus, this may contribute to serious thoracic injuries by allowing interior deformation to the occupant space in the event of a side impact. 

This study suggests that a higher crash severity affects the risk of thoracic injury in a side impact. In particular, the risk of thorax injury increased in CE2+ crashes. Similar to previous studies, crash severity was found to positively increase severe injuries in MVOs. Page et al. suggested that the increase in Delta-V estimated using energy equivalent speed (EES) calculation for crash severity is a significant factor in determining injury outcomes [17]. Higuchi used NASS-CDS data to suggest a risk curve for thoracic injury depending on tSAB deployment [41]. The difference between the two curves in low-speed crashes was small; however, the risk of thoracic injury in high-speed Delta-V crashes was statistically significant. In addition, a previous study reported that Delta-V and crash extent have a positive effect on AIS2+ thorax injury in side impacts [36].

In our research, the use of seatbelts and seat positions did not contribute to the prevention of thoracic injuries in side impacts. Some previous studies have reported that near-side impacts have a higher risk of serious injuries regardless of seatbelt usage [29,42]. This study showed similar results that the seatbelt usage of MVOs had no preventive effects reducing thoracic injuries in both near and far-side impacts. 

In the case of seating position, the risk of thoracic injury increased only for driver seat occupants during far-side impacts. Compared with the passengers, it is possible for drivers to have a higher probability of injury when engaged in contact with the interior components during an accident. Hallman et al. suggested that airbag deployment against a stationary occupant may not represent the greatest possibility of out-of-position (OOP) injury to the thorax from torso-side airbags [43]. However, the remaining far-side occupants mostly sustained injuries from the center console, instrument panel, or seat belts. In addition, far-side occupants were likely out of position owing to events preceding the side impact and/or being unbelted [44]. The current standard three-point seatbelt was initially designed to restrain MVOs from frontal crashes rather than side impacts. However, drivers are more likely to contact with the steering wheel, because of which they are generally at a higher risk of thoracic injuries. 

In this study, we analyzed the injuries details of thoracic-specific regions according to tSAB deployment using EMR data from the trauma center (Table 4). The results suggest that rib fractures and lung contusions (or lacerations) have a significantly higher incidence. This result is similar to that of previous studies; however, the incidence of thoracic injury was higher when tSABs were deployed [3,28]. Thus, we performed a logistic regression analysis to confirm that tSAB contributed to injury risk in both regions. 

However, tSABs were found to have no effects in preventing injuries in the thorax regions. Surprisingly, the deployed tSABs tended to increase the risk of both lung and rib fractures; however, the difference was not statistically significant. This leads to contradictory results from previous studies showing that tSABs may provide a slight benefit in terms of reduced thoracic skeletal MAIS2+ injuries [22]. On the contrary, our results showed that deployed tSABs may adversely affect thoracic AIS2+ injuries of MVOs engaged in a side impact. This may be relevant to studies in which SABs were associated with adverse events that lead to injuries [45,46]. However, it is necessary to be aware of the statistical results obtained using different datasets regarding the preventative effects of SABs. Nevertheless, an in-depth consideration is continuously needed as the possibility of tSAB may cause thoracic injuries rather than preventative affection. 

This study has several limitations. Similar to many previous studies, this study also used short-scaled numbered data to confirm the effectiveness of tSABs against thoracic injuries. It is difficult to comprehensively judge complex crash types with statistical significance. Therefore, further investigations using relatively large amount of data are required to cover the various conditions of actual field accidents. However, the data used in this study were limited to a single country. This may lead to slightly different results because there is a difference in the market share of the various manufacturers and models in each region. Moreover, a safety standards of vehicle crashworthiness may affect difference in preventing injuries in different regions as well. Therefore, it is necessary to gather interests in the crash dataset to evaluate the effectiveness of passive safety devices in preventing injuries. As regards statistics, this study did not adopt detailed variables on the actual collision speeds of cars at the time of an accident. This may affect the injury rate depending on the survival of the driver and passengers in case of tSAB deployments. However, statistical analyses are not necessarily conclusive. Conducting crash tests with the simultaneous high-tech study of the physics of the distribution of forces, stresses, and their effects on humans (assessment of damaging effects) is required to accurately evaluate the effectiveness of tSABs.

## 5. Conclusions

This study suggests that tSABs had no effect on preventing tAIS2+ injuries in side-impact crashes. Factors that increase the risk of tAIS2+ were found to be age over 65 years, near-side impacts, colliding with fixed objects, perpendicular crashes, and CE2+ crashes. Although the risk factors contributing to thoracic injury differed according to the near- and far-side impacts, tSABs did not prevent thoracic injuries. Instead, the chances of tAIS2+ injuries were found to be higher in MVOs involved in side-impact crashes with tSAB deployment. Moreover, the incidence of lung injury and rib fractures increased during tSAB deployment. As the study suggests that tSAB deployment had no effect on preventing thoracic injuries in side-impact crashes, further in-depth investigation is necessary from the perspective of vehicle crashworthiness.

## Figures and Tables

**Table 1:** Comparison of demographic characteristics according to thoracic side airbag deployments.

Variables	Total (n = 490)	tSAB Deployed (n = 40)	tSAB Undeployed (n = 450)	*p*-Value
Sex, n (%)				
Male	301 (61.4)	24 (60.0)	277 (61.6)	
Female	189 (38.6)	16 (40.0)	173 (38.4)	0.846
Age (mean±S.D.)	44.3 ± 16.3	40.1 ± 14.8	44.7 ± 16.4	0.084
Seat position, n (%)				
Driver	314 (64.1)	29 (72.5)	285 (63.3)	
Passenger	96 (19.6)	9 (22.5)	87 (19.3)	
2nd-row left passenger	35 (7.1)	1 (2.5)	34 (7.6)	
2nd-row right passenger	45 (9.2)	1 (2.5)	44 (9.8)	0.297
Seat belt, n (%)	n = 468	n = 40	n = 428	
Fastened	319 (65.1)	35 (87.5)	284 (66.4)	
Unfastened	149 (30.4)	5(12.5)	144 (33.3)	0.006
Vehicle type, n (%)				
Sedan	343 (70.0)	32 (80.0)	311 (69.1)	
SUV	105 (21.4)	7 (17.5)	98 (21.8)	
Van	42 (8.6)	1 (2.5)	41 (9.1)	0.245
Product year, n (%)	n = 297	n = 27	n = 270	
1996–1999	12 (4.0)	0 (0.0)	12 (4.4)	
2000–2004	59 (19.9)	0 (0.0)	59 (21.9)	
2005–2009	101 (34.0)	2 (7.4)	99 (36.7)	
2010–2014	95 (32.0)	16 (59.3)	79 (29.3)	
2015+	30 (10.1)	9 (33.3)	21 (7.8)	<0.001
Collide partner, n (%)	n = 418	n = 39	n = 379	
Movable	354 (84.7)	32 (82.1)	322 (85.0)	
Fixed	64 (15.3)	7 (17.9)	57 (15.0)	0.631
PDOF				
Perpendicular	203 (41.4)	20 (50.0)	183 (40.7)	
Oblique	287 (58.6)	20 (50.0)	267 (59.3)	0.251
Impact direction, n (%)				
Near-side impact	256 (52.2)	29 (72.5)	227 (50.4)	
Far-side impact	234 (47.8)	11 (27.5)	223 (49.6)	0.007
Crush extent, n (%)				
CE1 (1–2)	260 (53.1)	19 (47.5)	241 (53.6)	
CE2 (3–4)	213 (43.5)	17 (42.5)	196 (43.6)	
CE3 (5–6)	11 (2.2)	3 (7.5)	8 (1.8)	
CE4 (7–9)	6 (1.2)	1 (2.5)	5 (1.1)	0.087
AIS2, n (%)				
AIS<2	366 (74.7)	25 (62.5)	341 (75.8)	
AIS≥2	124 (25.3)	15 (37.5)	109 (24.2)	0.086
MAIS, median (IQR)	2 (1–3)	2 (1–3)	2 (1–3)	0.712
ISS, median (IQR)	5 (2–0)	5 (2–10)	4 (2–9)	0.931

Note: SAB: thoracic side airbags, SUV: sports-utility vehicle, PDOF: principal direction of force, AIS: Abbreviated Injury Scale, MAIS: maximum Abbreviated Injury Scale, ISS: injury severity score, S.D.: standard deviation, IQR: interquartile range.

**Table 2:** Multiple logistic regression analysis on thoracic AIS2+ injuries.

Variables	Unadjusted ORs (95% CI)	*p*-Value	Adjusted ORs (95% CI)	*p*-Value
***a. Overall side impacts***				
Age group (elderly)	1.76 (0.99–3.13)	0.055	2.96 (1.33–6.73)	0.008
Seat belt (fastened)	0.84 (0.54–1.30)	0.431	0.64 (0.34–1.20)	0.163
tSAB (deployed)	1.88 (0.96–3.69)	0.068	1.65 (0.73–3.73)	0.231
Seat position (driver)	1.40 (0.85–2.33)	0.190	1.32 (0.69–2.51)	0.400
Side impact (near)	2.88 (1.86–4.47)	< 0.001	4.30 (2.33–7.94)	< 0.001
Collide partner (fixed)	2.88 (1.66–4.99)	< 0.001	2.57 (1.26–5.26)	0.010
Impact direction (oblique)	0.44 (0.29–0.67)	< 0.001	0.43 (0.25–0.76)	0.004
Crush extent (CE2+)	3.86 (2.48–5.99)	< 0.001	4.85 (2.69–8.74)	< 0.001
***b. Near-side impact***				
Age group (elderly)	1.54 (0.71–3.34))	0.274	3.41 (1.12–10.36)	0.030
Seat belt (fastened)	0.93 (0.53–1.62)	0.787	0.53 (0.23–1.19)	0.124
tSAB (deployed)	1.65 (0.75–3.60)	0.211	1.70 (0.67–4.31)	0.265
Seat position (driver)	0.91 (0.49–1.72)	0.781	0.77 (0.35–11.71)	0.525
Collide partner (fixed)	3.13 (1.54–6.37)	0.002	2.95 (1.24–7.02)	0.015
Impact direction (oblique)	0.46 (0.27–0.78)	0.004	0.42 (0.26–0.84)	0.014
Crush extent (CE2+)	3.96 (2.29–6.86)	< 0.001	4.58 (2.27–9.21)	<0.001
***c. Far-side impacts***				
Age group (elderly)	2.41 (0.97–5.97)	0.057	3.56 (0.99–12.75)	0.051
Seat belt (fastened)	0.70 (0.33–1.49)	0.352	0.71 (0.24–2.07)	0.527
tSAB (deployed)	1.24 (0.26–5.97)	0.793	2.25 (0.38–13.16)	0.370
Seat position (driver)	2.57 (1.05–6.34)	0.040	4.27 (1.36–13.47)	0.013
Collide partner (fixed)	2.20 (0.84–5.79)	0.110	1.74 (0.43–7.08)	0.441
Impact direction (oblique)	0.37 (0.18–0.77)	0.008	0.45 (0.16–1.23)	0.119
Crush extent (CE2+)	4.85 (2.10–11.18)	< 0.001	6.15 (1.87–20.25)	0.003

Note: tSAB: thoracic side airbags, PDOF: principal direction of force, OR: odds ratio, CI: confidence intervals.

**Table 3:** Thoracic injury distribution according to tSAB deployment in side impacts.

Variables	Total (n = 490)	tSAB Deployed (n = 40)	tSAB Undeployed (n = 450)	*p*-Value
Diaphragm laceration/rupture				
Yes	4 (0.8)	0 (0.0)	4 (0.9)	
No	486 (99.2)	40 (100.0)	446 (99.1)	1.000
Lung contusion/laceration				
Yes	47 (9.6)	8 (20.0)	39 (8.7)	
No	443 (90.4)	32 (80.0)	411 (91.3)	0.020
Hemo-/Pneumothorax				
Yes	59 (12.0)	8 (20.0)	51 (11.3)	
No	431 (88.0)	32 (80.0)	399 (88.7)	0.107
Rib fracture				
Yes	99 (20.2)	14 (35.0)	85 (18.9)	
No	391 (79.8)	26 (65.0)	365 (81.1)	0.015
Sternum fracture				
Yes	8 (1.6)	2 (5.0)	6 (1.3)	
No	482 (98.4)	38 (95.0)	444 (98.7)	0.133

Note: tSAB: thoracic side airbags.

**Table 4:** Multiple logistic regression analysis associated with lung injury and rib fracture injuries (AIS ≥ 2) in side impacts.

Variables	Adjusted ORs (95% CI)	*p*-Value	Adjusted ORs (95% CI)	*p*-Value
	*a. Lung Contusion/Laceration*		*b. Rib Fractures*	
Age group (elderly)	2.22 (0.86–5.71)	0.100	1.54 (0.63–3.74)	0.334
Seat belt (fastened)	0.90 (0.40–2.06)	0.809	0.74 (0.37–1.49)	0.396
tSABs (deployed)	2.35 (0.92–6.03)	0.075	1.99 (0.85–4.67)	0.115
Side impact (near-side)	1.86 (0.86–4.01)	0.114	2.97 (1.51–5.85)	0.002
Seat position (driver)	1.40 (0.63–3.10)	0.408	1.12 (0.55–2.28)	0.753
Collide partner (fixed)	1.42 (0.57–3.51)	0.451	1.17 (0.52–2.60)	0.707
Impact direction (oblique)	0.62 (0.30–1.28)	0.194	0.46 (0.25–0.86)	0.015
Crush extent (CE2+)	3.50 (1.56–7.85)	0.002	5.07 (2.50–10.28)	<0.001

Note: tSAB; thoracic side airbags, CE; crush extent, OR; odds ratio, CI; confidence intervals.
